# Supplementary material for: Integration experiences of internationally educated diagnostic radiographers working in the UK
Source: PLoS One. 2025 Jun 11;20(6):e0325446. doi: 10.1371/journal.pone.0325446 (PMC12157326; doi:10.1371/journal.pone.0325446)
Supplement: S1 Table — (PDF) [file pone.0325446.s001.pdf]

S1 Table: Survey questions reported on in this article*

**Demographics & characteristics questions**	**Multiple choice responses**
What is your age range?	18-25 yrs old; 26-35 yrs old; 36-45 yrs old; 46-55 yrs old; 56-65 yrs old; >65 yrs old
Continent of origin (optional)	Australasia; Africa; Asia; Europe; North America; South America
Gender (optional)	Female; Male; Non-binary; Other; Prefer not to say
Marital status - current (optional)	Single; Married; Partnership/ Civil Union; Separated; Divorced; Widowed; Other; Prefer not to say
Marital status – before UK arrival (optional)	Single; Married; Partnership/ Civil Union; Separated; Divorced; Widowed; Other; Prefer not to say
Which term best describes your ethnicity? (optional)	Asian -Chinese; Asian- Indian; Asian – Pakistani; White and Asian; Any other Asian background; Black -African; Black –Caribbean; Black - British; White and Black African; Any other mixed or multiple ethnic background; Other; Prefer not to say
**Integrating into UK workplace statements (Likert Scale: Strongly agree; Agree; Neither agree nor disagree; Disagree; Strongly disagree)**	
I did not know what to expect when transiting to work in the UK	
I felt nervous adjusting to the work environment in my first few weeks	
I felt supported by my employer to address any anxiety I felt while joining my new job in the UK	
I was satisfied with the training/mentorship I received during my induction period.	
I felt the clinical practice in my UK job differed from the practice I had undertaken in my home country (e.g., with regards to examination protocols, departmental policies or work culture)	
I had the right clinical support from colleagues while adjusting to my new work environment.	
I had the right support from my line management while adjusting to my new work environment.	
I could easily identify a mentor/supervisor to approach with any work-related question I had during my induction.	
I was confident that I operated at the required competency level when I started my new job in the UK.	
I am comfortable with the radiography practice in the UK and unlikely I will be considering moving to a different country for a job.	
I feel supported to achieve my career aspirations as a radiographer.	
I experienced challenges communicating with colleagues and service users.	
I knew how to access any information necessary to work effectively in my job (e.g., policies, protocols, guidelines).	
I feel motivated to contribute to the service delivery and patient care in my department.	
I consider my work relationship with colleagues and managers to be cordial.	
I found it easy adjusting to the radiography workplace culture/system in the UK.	
I had a social network that I belong to that helped me adjust to life and work in the UK.	
My employer/ colleagues were a source of support that helped me adjust to life and work in the UK.	
I feel I have equality of opportunity for career progression and advancement in my UK role compared to other colleagues.	
I have experienced being bullied in the workplace as a result of my background as an international radiographer recruit.	
I considered my previous training and experience to have suitably prepared me for a radiography role in the UK.	

The knowledge/skills from my previous training and experience are respected and valued in my UK role.	
There were times I felt isolated after I started my role.	
I experienced differences in societal culture that affected my adaptation to life and work in the UK.	
Sometimes I feel that I do not "fit in" culturally within the team.	
**Reason/s which will help or helped your integration into UK workforce<br>(Ranking importance 1 to 5: 1 most being important)**	
Appropriate Work Mentoring	
Family Support	
Relocation Allowance	
Initial Housing Support	
Available training to meet clinical competency	
Available support to meet professional needs	
Good relationships in the workplace	
Effective communication in the workplace	
Socialisation and adapting to UK culture	
Motivation to work in the UK e.g., increased Salary	
Ease of recruitment and onboarding process	
Access to Information	
Line management support	
Easy adaptation to technology/ equipment's used in practice	
Other reason/s which will help or helped your integration into UK workforce<br>(free text response)	
**Intentions to remain questions**	**Response type**
Before you entered the UK, or immediately following arrival, was your intention?	Multiple choice options:<br>To stay for a short time before returning back to my home country;<br>To remain in the UK long term;<br>I was not sure
Any additional comments?	Free text response
Based on your experiences are you now intending to:	Multiple choice options:<br>To return to my home country;<br>To remain in the UK long term;<br>I am not sure
Any additional comments?	Free text response

* Other survey items have been reported in an earlier article [24]
