# Supplementary material for: Integration experiences of internationally educated diagnostic radiographers working in the UK
Source: PLoS One. 2025 Jun 11;20(6):e0325446. doi: 10.1371/journal.pone.0325446 (PMC12157326; doi:10.1371/journal.pone.0325446)
Supplement: Attachment — Submitted filename: Response to reviewers.docx [file pone.0325446.s003.docx]

Response to reviewers

The authors would like to thank the reviewer and editor for their value insight, suggestions for improvement and identifcation of omissions within our manuscript, all of which we have considered in our resubmission as outlined blow.

| **Reviewer comment** | **Response from authors** |
| --- | --- |
| Abstract - add year data collected | 2023 has been added |
| Abstract - add an aim or objective | Aim added to abstract |
| Introduction line 72 –rephase sentence for clarity | This has been separated into two sentences and reworded for clarity. |
| Introduction – explain why this focus is interesting | Thank you- additional rationale added to the introduction. |
| Method – year ethics obtained & by whom | Method line 78 wording has been updated to ‘Ethical approval was obtained from the Research Ethics Panel at the University of Bradford on 15^th^ September 2023 by the authors employed there prior to study commencement (EC27952).’ |
| Method - How many piloted the survey-and did that lead to any changes | Lines 88-91 have been amended to include the number of individuals involved in the pilot phase and to confirm that the changes related to question wording and survey routing. |
| Method – Was it possible to skip survey items | Results line 22 indicates respondents did not answer all questions but for clarity as you suggest, additional information has been given in the method and a copy of the survey questions has been added as supplementary information (S1 Table). |
| There seems to be many qualitative responses – did you perform a qualitative analysis as well as the quantitative calculations? Please explain. | Additional explanation added to methodology regarding thematic analysis of qualitative free text responses |
| Why collect data on ethnicity. I would think educational backgrounds were more important than ethnicity? | All data collection *was* undertaken in the UK as the population was internationally educated diagnostic radiographers already working in the UK. Ethnicity as well as country of origin was obtained to explore whether there was a correlation with transition or discrimination as has been reported in other research regarding migrant healthcare workers. Information on education was collected and reported in our already published paper, and we are reporting on different data sets from the survey in this manuscript as explained in method lines 95 & 96. |
| Consistency in terminology -terms such as “IEDR” | Terminology has been changed where appropriate to keep consistency throughout manuscript |
| To strengthen the research, it is important to clarify the statistical analysis by stating whether any missing data were handled using specific techniques or ignored on a case-by-case basis. | Missing data was considered on a case by case basis – this has now been reported in the method section. |
| Provision of data.  All PLOS journals now require all data underlying the findings described in their manuscript to be freely available to other researchers, either 1. In a public repository, 2. Within the manuscript itself, or 3. Uploaded as supplementary information. | Participant privacy in the use of the data was assured in the study information sheet to encourage participation, with information that the data be retained only by the research team. This approach and this wording on the participant information sheet was approved by the Universities Ethics Panel. Specific data is available from the authors on request. |
| Overall a nice discussion with relevant points for the UK. However, what about comparison with US or the Arab countries (Saudi, Abu Dhabi etc.)? Both countries recruit foreigners to healthcare. | Additional references have been added to give a global comparison to UK trends. |
| What about moral obligation to the countries recruiting foreigners – any thought about brain drain? | This is a worthy topic for discussion, however the focus of this paper is integration and experiences for those who have chosen to migrate rather than the issues migration creates. We have published an earlier paper related to a different set of data from the survey which maps where migrant radiographers come from to work in the UK and the drivers. This paper has been referenced in this article. |
| The conclusions could be expanded to better connect the findings with policy implications | This has been added to the conclusion, however, we have not touched on policy regarding international recruitment and its ethics for reasons stated above. |
| Limitations – was the validation of the survey limited? | We have added a sentence to the limitations confirming that we did not seek to validate the survey tool through a formal process but have acknowledged that this should be an aspiration to ensure its applicability going forwards. |
| Is this a limitation – consider to delete? ‘further research exploring the experiences of employers supporting the integration of IEDR into the 389 UK radiography workforce would be valuable and provide opportunity for triangulation of findings’. | This sentence has been removed from limitations and added to the implications for practice section |
| Check formatting of references | Amended to Vancouver with square brackets as per guidance |
